# Using Demographic Estimation and Power Analysis to Inform Monitoring Efforts and Detect Declines in Freshwater Mussels

**DOI:** 10.1002/ece3.73532

**Published:** 2026-05-14

**Authors:** Michael A. Baker, Allison H. Roy, Jason Carmignani, Brian J. Irwin, Clark Rushing, Sean Sterrett, Beth Swartz, Peter D. Hazelton

**Affiliations:** ^1^ Warnell School of Forestry and Natural Resources University of Georgia Athens Georgia USA; ^2^ U.S. Geological Survey, Massachusetts Cooperative Fish and Wildlife Research Unit University of Massachusetts Amherst Massachusetts USA; ^3^ Massachusetts Division of Fisheries and Wildlife: Natural Heritage and Endangered Species Program West Borough Massachusetts USA; ^4^ U.S. Geological Survey, Georgia Cooperative Fish and Wildlife Research Unit, Warnell School of Forestry and Natural Resources University of Georgia Athens Georgia USA; ^5^ School of Science Monmouth University West Long Branch New Jersey USA; ^6^ Maine Department of Inland Fisheries and Wildlife‐ Wildlife Diversity Section Bangor Maine USA

**Keywords:** *Alasmidonta varicosa*, brook floater, capture probability, mark‐recapture, population abundance, survival

## Abstract

Population assessments can be used to determine species' viability and inform conservation efforts; however, obtaining sufficient data quality and quantity can be challenging when monitoring resources are scarce. Brook Floater ( *Alasmidonta varicosa* ) is a freshwater mussel that is state listed in the United States as endangered or threatened in 10 of the 14 states it occupies. Despite the conservation concern, little is known about the size and demographics of most Brook Floater populations. The primary objective of this study was to determine demographic parameters (abundance and survival) for Brook Floater at five newly established, long‐term monitoring sites in Maine and Massachusetts, United States. A secondary objective was to evaluate tradeoffs of potential future sampling strategies by state natural resource agencies at these monitoring sites. Mussels were sampled at each site using visual and tactile snorkel surveys twice each summer during 2018–2020 and marked with alphanumeric tags to distinguish individuals. We considered a candidate set of capture‐recapture models that allowed parameters to vary depending on year, sampling occasion, and individual shell length. Abundance estimates among sites varied widely, from 29 individuals (range: 12–119) in the Nissitissit River in Massachusetts to 311 individuals (range: 307–320) in the East Branch of the Pleasant River in Maine. Annual survival was high across all sites (0.87–0.96). Given the differences in capture probability and abundance between states, we determined the statistical power available to detect given population declines in Massachusetts and explored alternative population abundance estimators in Maine. The power analysis suggested that the existing sampling regimen in Maine is likely adequate while sampling in Massachusetts may require additional occasions each year to realistically detect population declines with sufficient statistical power. This analysis demonstrates the utility of considering sampling frequency when designing capture‐recapture studies for freshwater mussels and other taxa.

## Graphical abstract

Population assessments can be used to determine species' viability and inform conservation efforts; however, obtaining sufficient data quality and quantity can be challenging when monitoring resources are scarce. This study combines field methods and data simulation to provide insight about population changes and the sampling necessary to detect those changes.

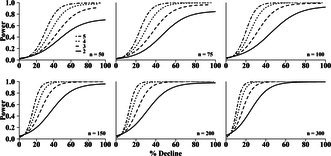

## 1 Introduction

Understanding population demographics is critical to species management and recovery (Warlick et al. 2022). For instance, estimation of population parameters such as species presence, abundance, survival, or movement rates is valuable for assessing population trends and protecting imperiled species (Campbell et al. 2002; McGowan et al. 2017). Further, data from monitoring programs are critical for parameterizing scenario forecasting models, which can make predictions about the state of a system in the future to inform critical management decisions (Irwin, Wilberg, et al. 2008). Observing biological processes and changes in demographic rates often requires multiple generations at the population scale (Rushing et al. 2016; White 2019; Haubrock et al. 2023). For example, describing how a system has responded to environmental stressors or disturbances (Irwin et al. 2016; Vidal et al. 2017) or quantifying the effectiveness of implemented management decisions and determining realistic sampling strategies to detect population level change (Bradke et al. 2023; Fromont et al. 2024) all require long‐term data.

To accomplish these management objectives above, repeated sampling (often referred to as long‐term monitoring) is frequently implemented. The scale of this monitoring is usually dependent on the life history traits and the generation time of the study organism (Lindenmayer et al. 2022). Monitoring of days or weeks may be considered long term when studying a short‐lived organism (e.g., many insect species), but even a 100‐year study may not be considered long term when researching long‐lived species of trees or mollusks (Lindenmayer and Likens 2018). Periodicity of repeated sampling can also be highly variable. Some natural resource managers may have the resources to continuously sample a site each year, but others may be more constrained, resulting in an intermittent sampling approach (e.g., a few years each decade). In either case, effective long‐term monitoring involves not only the tracking of changes in populations or parameters of interest but also an understanding of management actions available depending on the changes detected (Gitzen 2012). While the costs associated with maintaining long‐term monitoring studies are sometimes prohibitive (McDonald‐Madden et al. 2010), the benefits of long‐term monitoring have been thoroughly demonstrated (Lindenmayer et al. 2022). In some cases, data from multiple surveys can be combined to develop a more complete description of how a population has changed over time (Irwin, Treska, et al. 2008).

Freshwater mussels within the order Unionida are a taxonomic group for which long‐term monitoring may be particularly necessary to obtain insights into population dynamics and demography. Found in lentic and lotic systems worldwide, freshwater mussels are among the most imperiled species groups globally (Lydeard et al. 2004). Their sessile nature, slow growth, late maturation, and long lifespans (i.e., up to several decades) result in populations that must be repeatedly sampled over an extended period (i.e., multiple years or even decades) to obtain information about demographics and other population‐level changes that are not generally possible to obtain with single season studies (Sansom et al. 2018; Hopper et al. 2024). Long‐term monitoring has been used to estimate demographic rates in freshwater mussels (Kesler et al. 2007; Ries et al. 2016; Lane et al. 2021); however, most examples of long‐term monitoring in the context of freshwater mussels have specifically described and tracked declines (as opposed to growth), either at the population or assemblage level (Hornbach et al. 2017; Gonzalez et al. 2021; Lopez et al. 2022; Nakamura et al. 2023).

The primary goal of this study was to understand the population demographics for an imperiled freshwater mussel species, the Brook Floater ( *Alasmidonta varicosa* ), at several newly established long‐term monitoring sites in the northeastern United States. The specific objectives were to (1) use mark‐recapture approaches to estimate annual abundance, survival, and capture/recapture probabilities of Brook Floater at five sites, and (2) evaluate tradeoffs of potential future sampling approaches by state natural resource management agencies at these sites. The results will help develop realistic expectations about continued monitoring efforts at these sites. While our data are site‐specific estimates, evaluation of sampling tradeoffs can guide agency managers in the prioritization of resources for other long‐term monitoring programs.

## 2 Methods

### 2.1 Study Species

The Brook Floater ( *Alasmidonta varicosa* ) is a species of freshwater mussel that primarily inhabits medium‐sized streams and rivers and is considered to be a habitat specialist (Nedeau et al. 2000; Nedeau 2008; Haag 2012; U.S. Fish and Wildlife Service [USFWS] 2018). While little information is available about specific habitat preferences, they are generally found in sand and gravel substrates in systems with moderate flow (Nedeau et al. 2000). Typically reaching an adult size of 60–80 mm, Brook Floater have relatively short life spans (12–15 years; Haag 2012). Although the species is broadly distributed across the east coast of North America from the Chattooga River in Georgia, United States to the Miramichi River in New Brunswick, Canada, Brook Floater is considered state listed as either threatened or endangered in 10 of the 14 states where it persists (Wicklow et al. 2017; USFWS 2018).

### 2.2 Study Area and Sites

**FIGURE 1:**
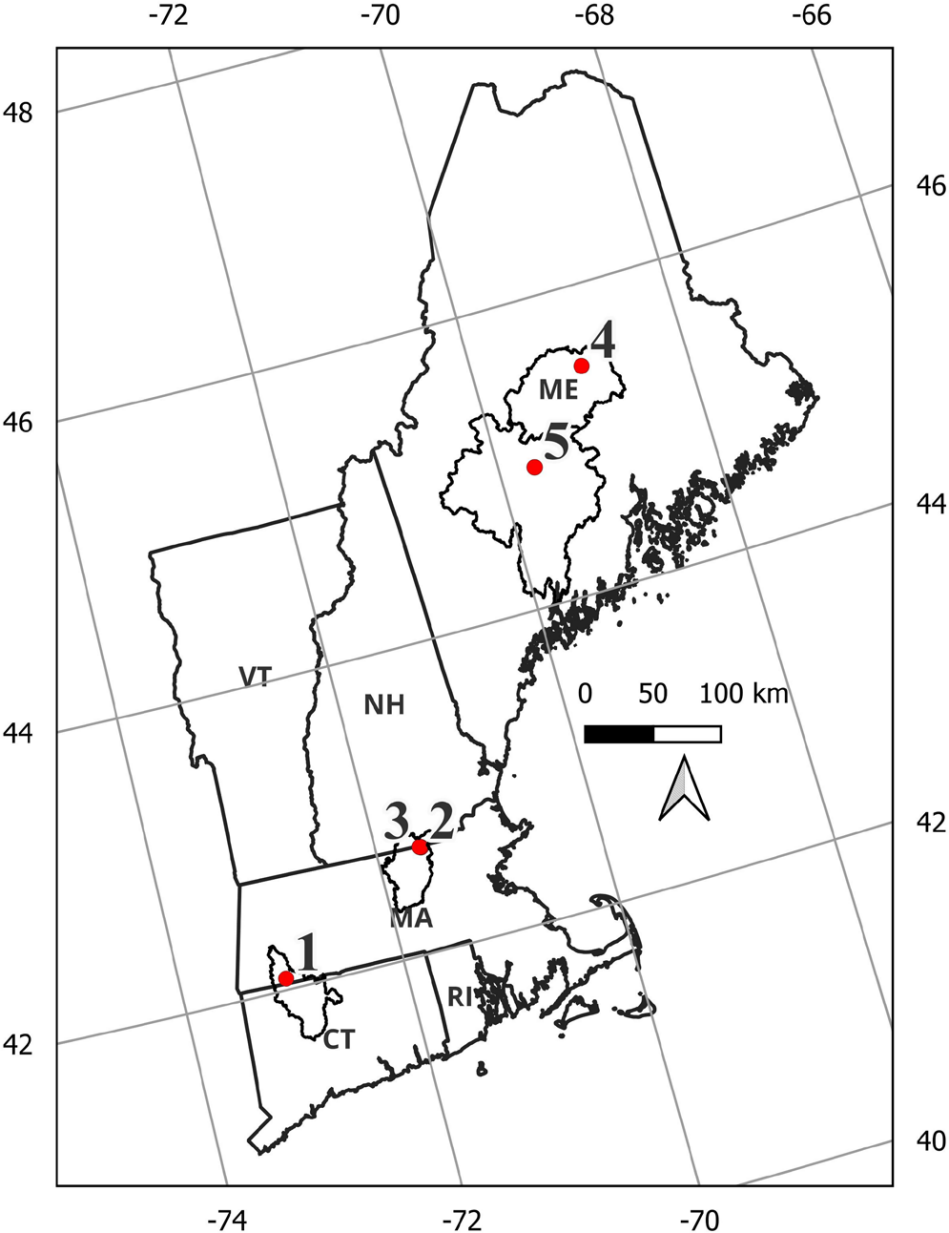
Map of New England (USA) showing Hydrologic Unit Code 6 (HUC6) level watersheds and locations of long‐term monitoring sites (1 = Farmington River; 2 = Old Dam [Nissitissit River]; 3 = Sucker Brook [Nissitissit River]; 4 = Pleasant River; 5 = Wesserunsett Stream).

Five sites were identified for this project as part of a range‐wide conservation initiative (Sterrett et al. 2022), three in Massachusetts and two in Maine, representing some of the northernmost habitats of Brook Floater (Figure 1). In Massachusetts, the species is state‐listed as endangered and is patchily distributed, currently known to occur in only four watersheds (Massachusetts Division of Fisheries and Wildlife [MDFW] 2019). Two sites (Old Dam and Sucker Brook) were selected in the Nissitissit River, a 16.9‐km‐long tributary of the Nashua and Merrimack rivers, which harbors a small population of Brook Floater (Marchand 2015). Both sites were located within a few river km upstream of the Millie Turner Dam, which was removed in 2015 (Abbott et al. 2022). In part due to the dam removal, the Nissitissit population is thought to have the opportunity to increase in abundance over the coming years (Wicklow et al. 2017; Abbott et al. 2022). The third Massachusetts site was in the West Branch of the Farmington River (hereafter, Farmington). Although considered one of the most biologically diverse rivers in New England, previous work there found that the population is skewed toward larger individuals (theoretically due to a lack of recent recruitment), raising concern for an impending population decline if recruitment does not occur (Nedeau and Low 2008). The specific reaches were chosen based on surveys indicating relatively high abundance of Brook Floater or, in the case of the Nissitissit River sites, areas where Brook Floater were translocated prior to the dam removal. In Maine, Brook Floater is found in over 30 rivers and streams in the mid‐coast, central, and eastern portions of the state and is currently state‐listed as threatened (USFWS 2018; MDIFW 2025). The sites in Maine were intended to represent a difference in perceived land‐use pressures with one site (East Branch of the Pleasant River; hereafter Pleasant River) in a more remote, primarily forested and undeveloped landscape and the other (Wesserunsett Stream) characterized by farmland and low density rural development. The Pleasant River hosts a large population (> 1000) of Brook Floater and is considered one of the most stable in the state (Wicklow et al. 2017). The Wesserunsett Stream hosts a stable population (likely in the 1000's) across a 20‐km stretch and has been the focus of prioritized survey efforts (Wicklow et al. 2017).

### 2.3 Mussel Sampling

All freshwater mussel sampling activities were permitted by the Maine Department of Inland Fisheries and Wildlife (MDIFW) and the Massachusetts Division of Fisheries and Wildlife (MDFW). Mussels were sampled twice per summer (June–August) over 3 years (2018–2020). Surveys consisted of timed searches within each delineated site (Table 1) using visual and tactile snorkel methods following the protocols of Sterrett et al. (2022). To ensure complete coverage, each site was divided into multiple observer lanes, which oriented along the flow path and usually extended bank to bank, and each observer was responsible for searching the substrate while moving upstream at a targeted maximum search rate of 10 m^2^/min (Sterrett et al. 2022). When searching through softer substrates, observers opportunistically excavated the substrate to a depth of up to 10 cm; in larger substrate, observers felt around boulders and cobble to detect mussels that may not be noticed visually. At one site, the Pleasant River, the observer lanes did not extend bank to bank due to funding limitations for sampling, so only the sampled area was considered part of the site. During each individual survey, observers were randomly assigned to each lane to prevent bias due to differences in observer experience. Detected mussels were flagged during searches using either pin flags or washers with flagging attached and later collected from the substrate after the survey was complete. Each mussel was identified to species and double tagged (one on each valve, to account for tag loss) using alpha numeric vinyl tags (1.0 cm long × 0.4 cm wide shellfish tags FT‐LF‐97; Floy Tag & Manufacturing, Seattle, WA) affixed with cyanoacrylate glue (Loctite Ultragel Control, Henkel Corporation, Rocky Hill, CT). Tag numbers were recorded for already‐tagged mussels, and lost tags were replaced. Shell length along the anteroposterior axis of each individual was recorded to the nearest millimeter using vernier calipers. Differences in mean shell length across sites were estimated using an analysis of variance (ANOVA) and a subsequent Tukey multiple comparisons test. After completion of the survey, all mussels were randomly placed back into the sampling area in a natural siphoning position.

**TABLE 1:** Site descriptions of the five long term monitoring sites where Brook Floater ( *Alasmidonta varicosa* ) were captured.

	Massachusetts			Maine	
	Farmington River	Nissitissit River Old Dam	Nissitissit River Sucker Brook	Pleasant River	Wesserunsett Stream
Width (m)	11	18	17	26	16
Length (m)	120	100	100	14.3	100
# Lanes	4	4	4	5	3
Area sampled (m^2^)	1320	1800	1700	375	1600

### 2.4 Data Analysis

#### 2.4.1 Objective 1: Estimation of Brook Floater Populations

Yearly survival, capture/recapture probability, and abundance were estimated using Jolly Seber models with a robust‐design approach implemented in the ‘RMark’ package of Program R (Version 3.4.2; Jolly 1965; Seber 1965; Pollock 1981; Pollock 1982; Williams et al. 2002; Laake 2013; R Core Team 2021). The robust design works by combining the benefits of both open and closed population models. Among each year of sampling (primary periods), the population is assumed to be open to births/deaths as well as individual immigration/emigration. Among sampling occasions within a particular year (secondary periods), the population is assumed to be closed to these gains or losses (Pollock 1982). As a result, demographic parameters can be estimated that otherwise could not be by using either an open or closed population model alone. Several other assumptions related to the populations of interest are also made when using these models (see Appendix S1). Briefly, the two assumptions that most affected sampling methods and model interpretations are: (1) the entire population of interest is available for capture and (2) no tags are lost during the study. To meet the first assumption, we only estimated site‐specific abundance for individuals ≥ 20 mm shell length since animals smaller than that size are rarely detected using snorkel surveys alone (Smith et al. 2001; for further explanation, see Appendix S1). Additionally, all animals were double tagged to reduce the effects of tag loss (see Appendix S1).

A candidate set of 72 models was constructed for each of the five sites to estimate survival, capture/recapture probability, and site‐specific abundance. Each model differed by either holding parameters constant or allowing the parameters to vary by year, sampling occasion, and shell length of the individual (Table 2). The candidate models were then ranked using Akaike's information criterion adjusted for small sample size (AIC_c_), and an AIC weight was calculated to determine the best model in the candidate set (Akaike 1973; Burnham and Anderson 2002). Confidence intervals for all estimated parameters were derived from the model‐produced standard errors (Burnham and Anderson 2002).

**TABLE 2:** Descriptions of model parameters included in the candidate set to estimate abundance of Brook Floater ( *Alasmidonta varicosa* ) at five long‐term monitoring sites from 2018 to 2020.

Parameter	Scenario	Description
Survival (ϕ)	Constant	Survival probability constant
	Year	Survival probability varies across primary periods (2018–2020)
	Length	Survival probability varies by individual shell length
Capture (c)	Constant	Capture probability constant
	Year	Capture probability varies across primary periods (2018–2020)
	Length	Capture probability varies by individual shell length
	Occasion	Capture probability varies across secondary periods
Recapture (*r*)	Shared	Recapture probability shared with capture probability
	Constant	Recapture probability not shared with capture probability
Movement	None	No temporary emigration occurs
	Random	Temporary emigration occurs; the probability of moving from one availability state to another between primary occasions does not depend on previous state
	Markovian	Temporary emigration occurs; the probability of moving from one availability state to another between primary occasions is conditioned on previous state

*Note:* Each model allows for different sources of variability in survival (ϕ), capture probability (c) and recapture probability (*r*) as well as different movement scenarios. Each scenario was considered for a given parameter as well as additive effects of applicable variables.

A goodness of fit test was performed using the ‘R2ucare’ package in Program R to evaluate the most general model (global model; Gimenez et al. 2018; R Core Team 2021). In theory, if this model reasonably fits the data, then it can be compared to all other reduced parameter models. Additionally, the variance inflation factor ($\hat{c}$) was calculated to assess the level of overdispersion in each model. A $\hat{c}$ ≤ 3 was considered adequate (Lebreton et al. 1992).

#### 2.4.2 Objective 2: Assessing Alternative Sampling Strategies

We identified potential future sampling strategies for estimating abundance via discussions with both state natural resource management agencies (MDFW and MDIFW). Our objective was to explore how the agencies' existing strategy might be modified to allow for the lowest use of sampling resources while still obtaining accurate and precise estimates of abundance. Given the differing results in capture probability and abundance as well as the precision of those estimates between the two states, two different approaches were used. For Maine, we explored simplified estimators of abundance, whereas in Massachusetts, we conducted a power analysis to determine how much additional sampling may be necessary to obtain reasonably precise estimates. These methods are described in more detail below.

#### 2.4.3 Simplified Estimators (Maine)

While the robust‐design framework is a commonly used approach to estimating several demographic parameters, other estimators are available for obtaining values of abundance while reducing the overall required sampling effort. One such estimator, the Lincoln‐Petersen Index, can be used to estimate yearly abundance using only two sampling occasions (Petersen 1896; Lincoln 1930) and can be particularly appealing when capture probabilities at all sampling events are high. This method has an advantage over the robust design in that it does not require three consecutive years of sampling to obtain a yearly abundance estimate. To anecdotally evaluate the usefulness of this method for estimating yearly abundance at the sites in Maine, we compared abundance estimates obtained from a robust‐design approach to those obtained using the Lincoln‐Petersen Index on the same data set.

#### 2.4.4 Power Analysis (Massachusetts)

A power analysis using a simulated dataset was conducted in Program R to assess the effects of various parameters on robust‐design model accuracy and precision (using the null model [M_0_]) and to determine whether model estimates were sensitive to additional sampling (beyond that already conducted by MDFW). Statistical power refers to the probability of detecting an effect given that it is there. In this context, we were interested in estimating the probability of detecting various percentages of population decline (across the range of 0%–100% decline) and quantifying the changes in power based on number of secondary periods. For example, if a 50% decline occurred, we sought to determine our statistical power to detect that a decline of that magnitude had occurred using a subsequent 3‐year sampling regimen after the decline took place. To do this, input parameters were chosen including initial population size (*n* = 50–300), number of primary (3) and secondary (2–5) periods, yearly mean survival probability (ϕ = 0.9), and mean capture probability (*p* = 0.3). Values for survival and capture probability were representative of sites found in Massachusetts during this study (Objective 1). A capture history for each individual in the population was constructed by first assigning yearly survival using the probability above and then simulating captures for years in which the individual was still alive. All simulated individuals that were never captured were removed from the capture history before proceeding. In doing so, we allowed the model to estimate the number of animals never observed, which was then used to determine the total population size. This process was repeated 1000 times, such that each iteration represents the capture histories for each 3‐year sampling study of the simulated population.

A robust‐design model was used to estimate the yearly population size for each of the 1000 iterations. Then, because the true simulated population size was known, we subtracted the true population size from the estimated population size at the end of the three‐year sampling and converted this to a percentage to obtain a metric of model error for a particular iteration. Positive values represent that the model had overestimated the population size for that iteration while negative values represent an underestimation. For each simulation, we checked if the confidence interval around the estimated population size contained the known size of the simulated population at the end of the projected period (i.e., year 3) and used the proportion of simulations for which this was true to compute the power to detect the specified population decline.

## 3 Results

**FIGURE 2:**
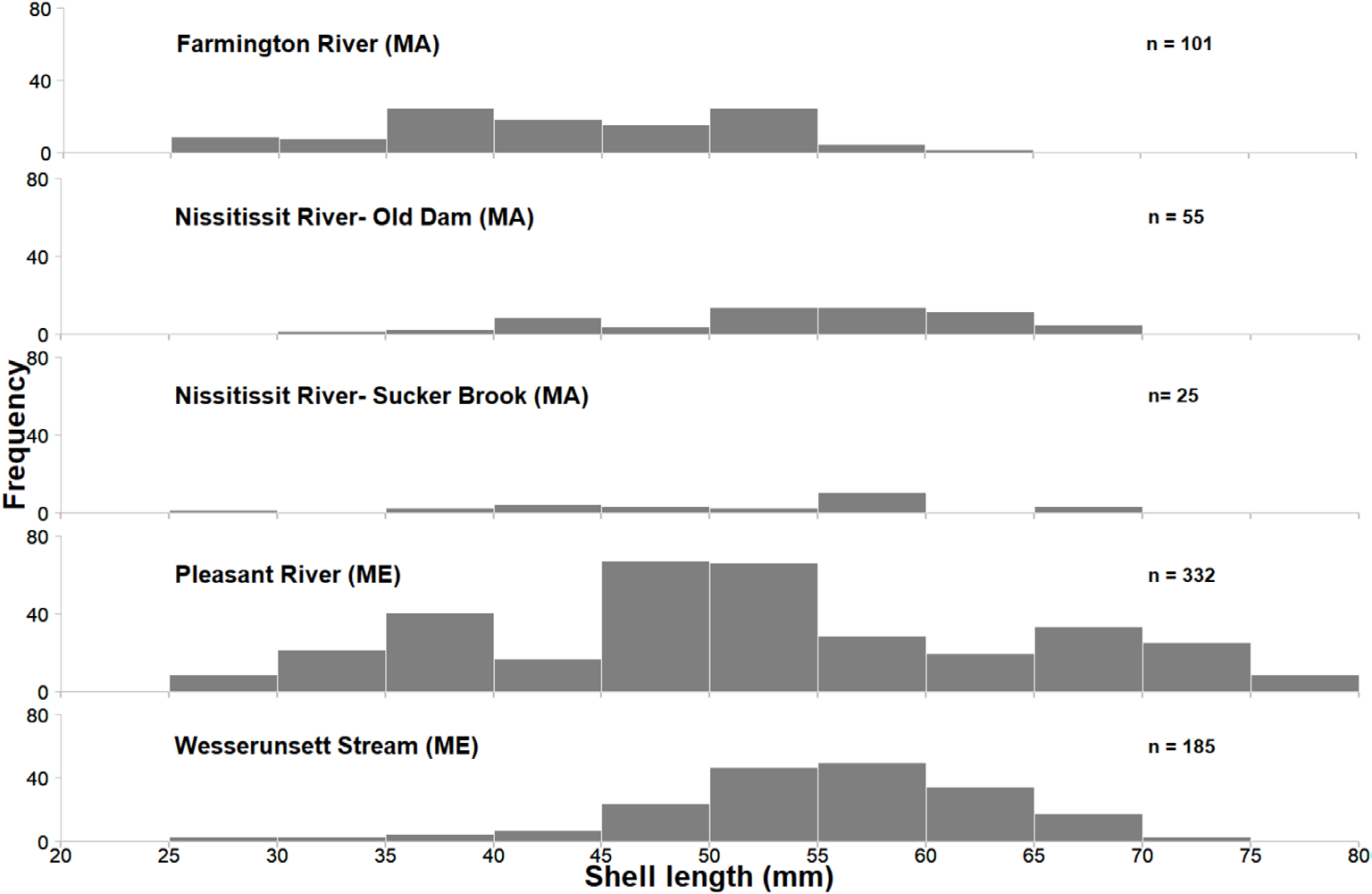
Length frequency histograms of Brook Floater ( *Alasmidonta varicosa* ) captured at five long‐term monitoring sites during the summers of 2018–2020. Sites in Massachusetts and Maine are represented by (MA) and (ME), respectively.

Sites ranged from 14.3 to 120 m in length and varied in area sampled from 375 m^2^ (Pleasant River, Maine) to 1800 m^2^ (Old Dam site, Nissitissit River, Massachusetts; Table 1). We captured and tagged 698 individual Brook Floater across the five sites (Table 3). Of those, 565 were recaptured at least once (with 25.5% being captured in all six sampling occasions), resulting in an overall recapture rate of 70.3% and a total of 2348 individual capture events. Captured Brook Floater exhibited a range of shell lengths (25.1–78.6 mm), with a mean shell length of 51.9 mm (Figure 2). Mean shell length of captured individuals was similar across sites, except the Farmington River site where mean shell length (43.5 mm) was significantly smaller than in all other sites (*p* ≤ 0.05). While recruitment was not formally estimated in the models, juvenile mussels were captured at all five sites (3%–9% of captured individuals at each site were ≤ 30 mm), suggesting the existence of recruitment.

**TABLE 3:** Number of individual Brook Floater ( *Alasmidonta varicosa* ) captured and recaptured from 2018 to 2020 at the five sites.

Site (State)	Year	New Captures	Recaptures	Total Individuals
Farmington River (MA)	2018	43	NA	101
	2019	40	18	
	2020	18	39	
Nissitissit River (MA) Old Dam	2018	32	NA	55
	2019	19	14	
	2020	4	30	
Nissitissit River (MA) Sucker Brook	2018	8	NA	25
	2019	10	3	
	2020	7	9	
Pleasant River (ME)	2018	256	NA	332
	2019	43	232	
	2020	33	270	
Wesserunsett Stream (ME)	2018	149	NA	185
	2019	26	130	
	2020	9	128	

*Note:* Total individuals represents the total number of captured individuals at that site. Sites in Massachusetts and Maine are represented by (MA) and (ME), respectively.

Abbreviation: NA, not applicable.

### 3.1 Objective 1: Estimation of Brook Floater Populations

The highest ranked model based on AIC values had similar predictor variables across all five sites where the best predictors included constant survival and shared recapture probability (Table 4); however, AIC weights suggested that multiple models within the candidate set were plausible (i.e., ∆AIC_c_ ≤ 2). Variation in survival due to year or length of individual did appear in other lower ranked models. Capture probability increased with shell length at nearly all sites (except at the Old Dam site on the Nissitissit River where it was constant; Table 4). In all of the highest ranked models, capture and recapture probability were shared, indicating the lack of a behavioral effect (Pollock 1982). Additionally, none of the top models included temporary emigration (i.e., $\gamma^{\prime }$ = $\gamma^{\prime \prime }$ = 0). A goodness‐of‐fit test resulted in no strong evidence of lack‐of‐fit (*p* > 0.05), and all variance inflation factor values within the top models for each site were estimated to be < 1.5, suggesting no significant overdispersion.

**TABLE 4:** Robust‐design models used to estimate abundance of Brook Floater ( *Alasmidonta varicosa* ) at five long term monitoring sites from 2018 to 2020.

Site (State)	Survival (ϕ)	Capture (c)	Recapture (*r*)	∆AIC_c_	*W*	*K*
Farmington River (MA)	Constant	Length	Shared	0.00	0.19	3
	Constant	Occasion + Year + Length	Shared	0.21	0.17	6
	Year + Length	Occasion + Year + Length	Shared	0.49	0.15	8
	Year	Occasion + Year + Length	Shared	1.01	0.12	7
	Year + Length	Occasion + Year	Constant	1.83	0.08	8
Nissitissit River (MA) Old Dam	Constant	Constant	Shared	0.00	0.15	2
	Constant	Occasion + Year	Constant	1.18	0.09	6
	Constant	Constant	Constant	1.20	0.08	3
	Constant	Occasion + Year	Shared	1.23	0.08	5
Nissitissit River (MA) Sucker Brook	Constant	Length	Shared	0.00	0.24	3
	Constant	Occasion + Year + Length	Shared	1.27	0.13	6
Pleasant River (ME)	Constant	Length	Shared	0.00	0.22	3
	Year	Length	Shared	0.52	0.17	4
	Year	Occasion + Year	Constant	1.19	0.12	8
	Year + Length	Occasion + Year	Constant	1.82	0.09	9
	Length	Length	Shared	1.82	0.09	4
Wesserunsett Stream (ME)	Constant	Length	Shared	0.00	0.72	3
	Year	Length	Shared	1.91	0.28	4

*Note:* Each model allows for different sources of variability in survival (ϕ), capture probability (c) and recapture probability (*r*) through covariates including primary period (Year), secondary period (Occasion), shell length (Length; mm), and capture status (Behavior). Differences in AIC_c_ (ΔAIC_c_), Akaike weights (*W*) and number of parameters (*K*) are given for each model. For brevity, only models with ∆AIC_c_ ≤ 2 are shown for each site. Sites in Massachusetts and Maine are represented by (MA) and (ME), respectively. Information on how recapture rates are incorporated into models can be found in Appendix S1.

**FIGURE 3:**
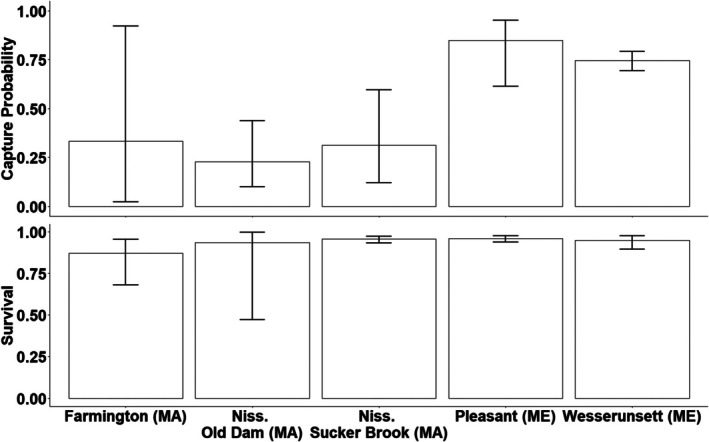
Mean estimated capture probability and yearly survival (with 95% confidence intervals) of Brook Floater ( *Alasmidonta varicosa* ) during 2018–2020 at five long term monitoring sites in Massachusetts (MA) and Maine (ME). Site names have been abbreviated for brevity.

**FIGURE 4:**
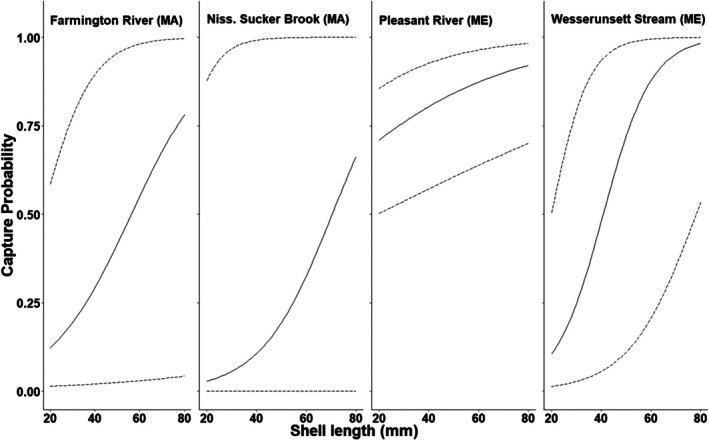
Capture probability (with 95% confidence bands) of Brook Floater ( *Alasmidonta varicosa* ) as a function of shell length (mm) at each of the four sites in Massachusetts (MA) and Maine (ME) where length was a significant predictor of capture probability in the top performing models during 2018–2020.

**FIGURE 5:**
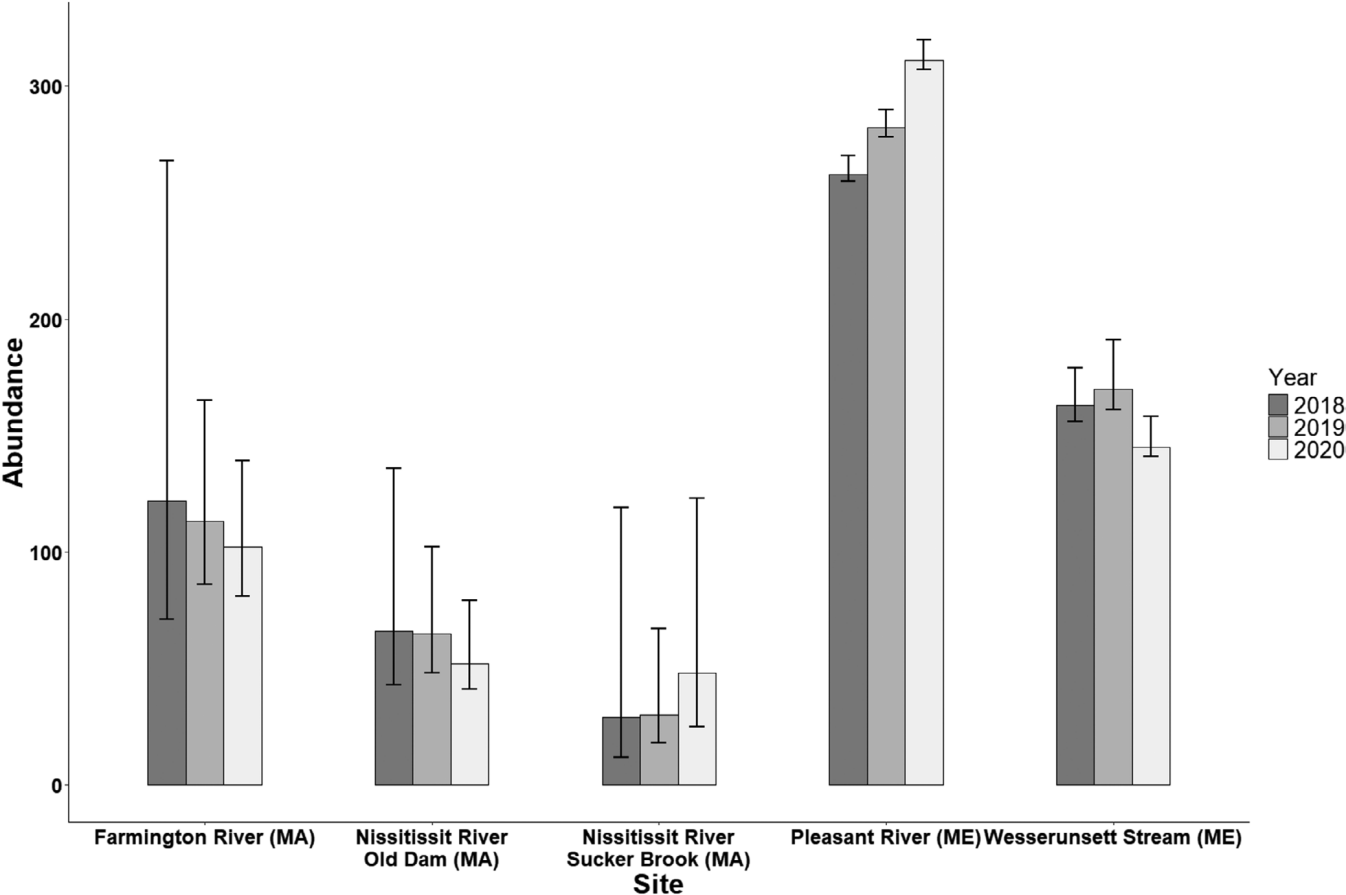
Abundance estimates (with 95% confidence intervals) of Brook Floater ( *Alasmidonta varicosa* ) derived from robust design method at five long term monitoring sites in Massachusetts (MA) and Maine (ME) during 2018–2020.

The best model from the candidate set was used to estimate the demographic parameters of interest for each of the five sites. Survival across all sites was high (mean = 0.93; 95% confidence interval [CI]: 0.90–0.96) with no significant differences among sites (Figure 3). Capture probabilities varied greatly among sites, with sites in Massachusetts being considerably lower (mean = 0.29; 95% CI: 0.23–0.35) than those in Maine (mean = 0.80; 95% CI: 0.70–0.90; Figure 3). At some sites, capture probability was positively correlated with shell size (Figure 4). Site‐specific abundance estimates also varied greatly across sites, ranging from 29 individuals in the Nissitissit River, Massachusetts, to 311 individuals in the Pleasant River, Maine (Figure 5).

### 3.2 Objective 2: Assessing Alternative Sampling Strategies

**FIGURE 6:**
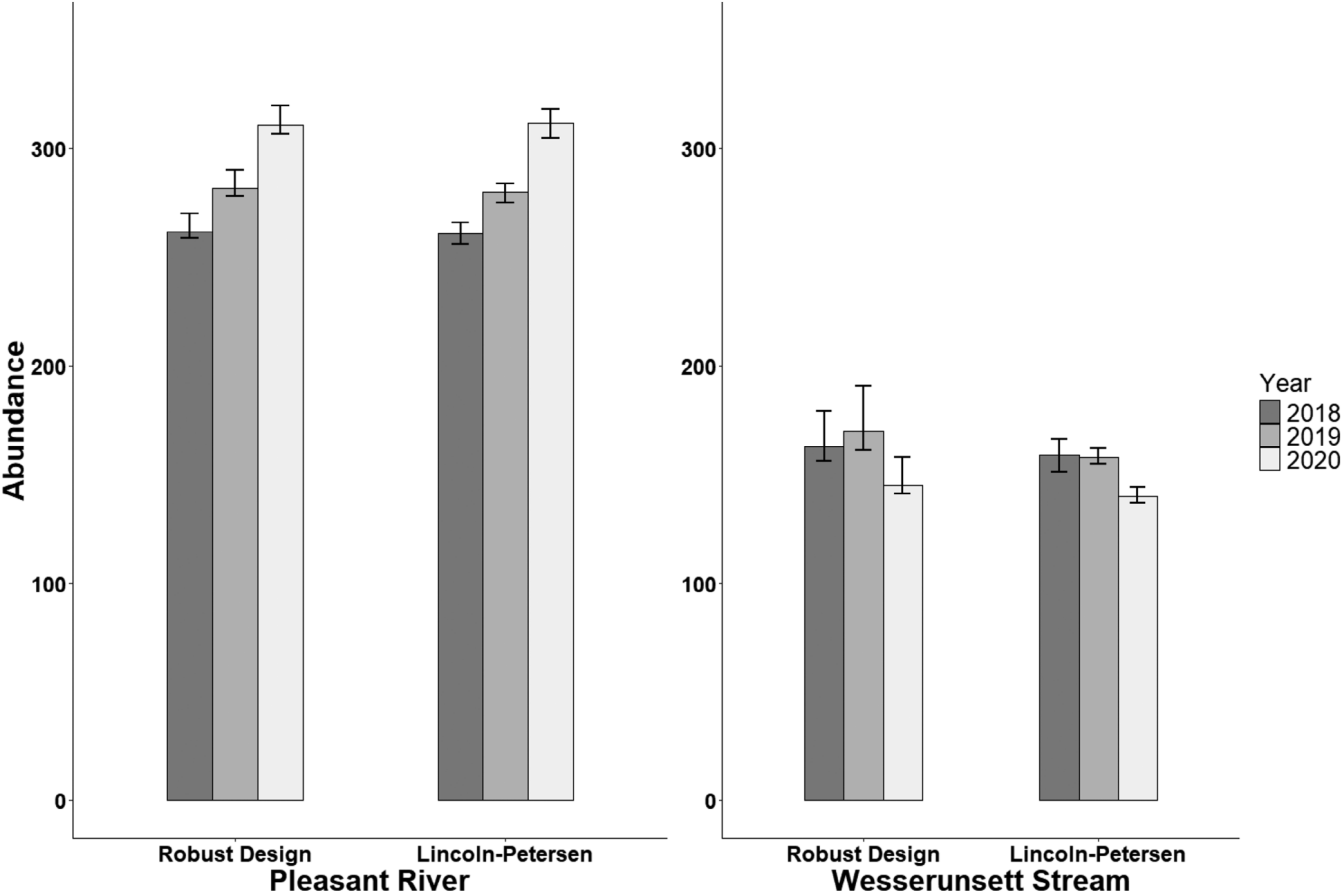
Abundance estimates (with 95% confidence intervals) of Brook Floater ( *Alasmidonta varicosa* ) in the Pleasant River and Wesserunsett Stream during 2018–2020 using the robust design and the Lincoln‐Petersen Index.

For each year of sampling at both sites in Maine, the Lincoln‐Petersen Index resulted in abundance estimates comparable to those derived from a robust‐design model (as demonstrated by overlapping confidence intervals; Figure 6). The Lincoln‐Petersen Index does not provide estimates of survival or capture probability since it is a closed population model.

**FIGURE 7:**
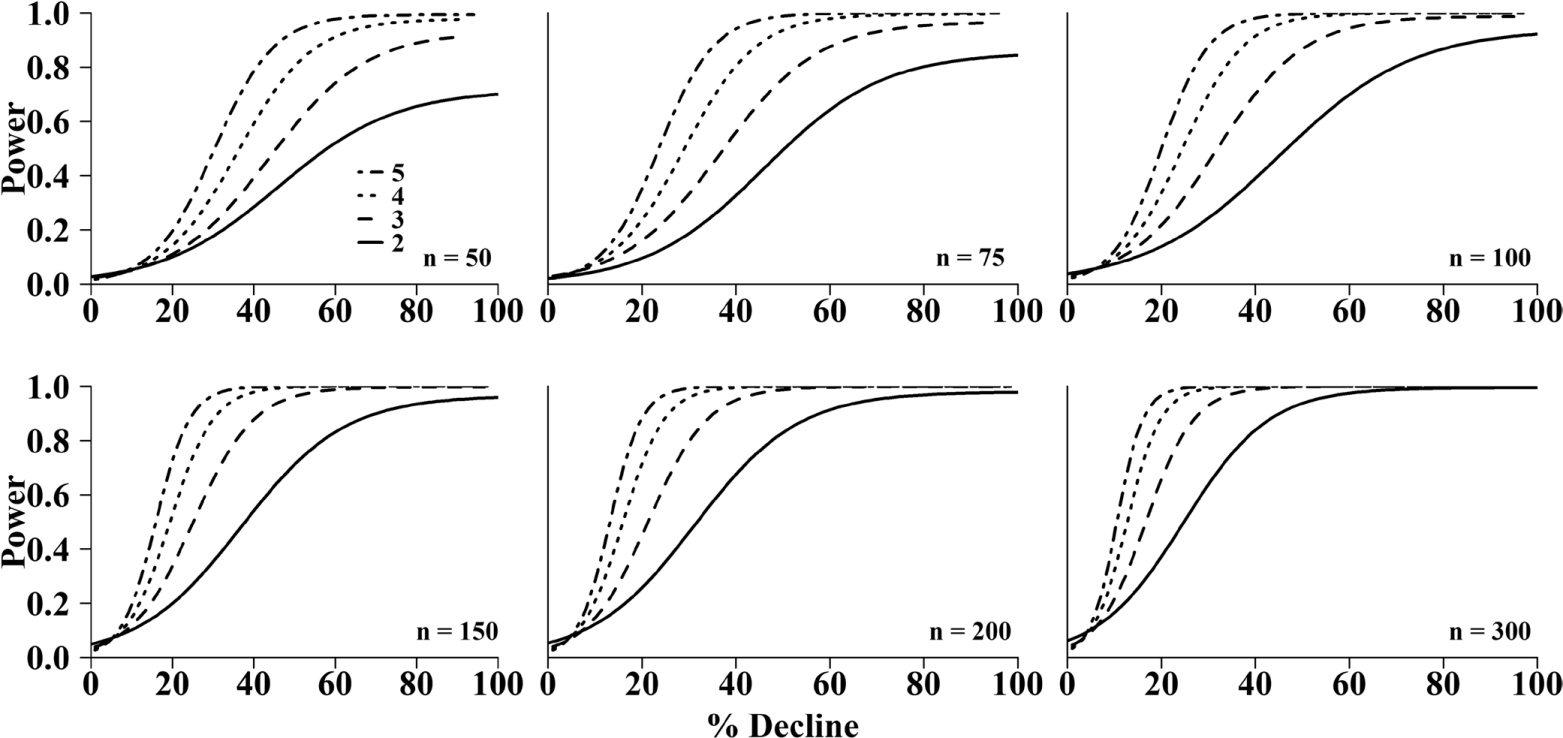
Power analysis used to show change in statistical power to detect a population decline (%) based on population size (n) and number of secondary periods (dashed lines) in a robust‐design framework. Input values included number of primary periods (3), survival (0.9), and capture probability (0.3).

The power analysis used to estimate our ability to detect a decreasing population demonstrated that increasing the number of secondary periods during each year of sampling would result in higher statistical power across all population sizes (Figure 7). In most scenarios (*n* ≥ 100), having three secondary periods instead of two resulted in an increase in power of ~30% when attempting to detect a 20%–40% decline. Additionally, in larger populations (*n* = 300; when using 4–5 secondary periods), power to detect population declines remained high, even for declines smaller than 20%. While two secondary periods may be sufficient to reach the conventionally agreed upon level of 0.8 for large populations, power does not exceed 0.8 until four or five secondary periods are implemented when considering small populations (< 150 individuals) or when the decline is 20%–40%. In some extreme cases, when the population is very small (< 50 individuals), even five secondary periods may not be sufficient for power to exceed 0.8 (Figure 7).

## 4 Discussion

Monitoring data plays a key role in understanding freshwater mussel population dynamics. While these sampled areas really represent portions of the larger populations within each of their respective river systems, defining the bounds of the monitoring sites allows natural resource managers to track changes at those locations over time. Our baseline data on Brook Floater abundance and survival at these five sites can be used to quantify population‐level changes from future restoration activities or assess environmental disturbances to track long‐term trends in those populations. Additionally, repeated sampling allows researchers to explore methodological questions that can improve future sampling efforts in these and other study systems.

### 4.1 Estimating Demographic Parameters Through Capture‐Recapture

The robust‐design framework for capture‐recapture implemented in this study is a useful tool when monitoring fish and wildlife populations, especially those persisting at low densities (Carey et al. 2019). This method allows for the estimation of several biologically meaningful parameters (survival, capture probability, abundance) while maintaining flexibility in the underlying assumptions made.

Survival was estimated to be generally high at all five sites, suggesting stable populations (although inference is limited given the short duration of the study). In contrast to other studies (Villella et al. 2004; Meador et al. 2011), survival did not vary with shell size in any of the top models. These prior studies used multi‐model averaged parameter estimates, an approach that has recently received criticism (Cade 2015), whereas the current study used only the estimates from the highest ranked model for each site. Additionally, the relatively small maximum size of this species and the fact that few individuals < 30 mm were captured meant that the range of shell sizes may have been too small to observe effects of size on survival. Alternatively, the short timeframe of the study (only 3 years) may have resulted in non‐detections of individuals being interpreted by the model as imperfect detection rather than lower survival; as with any similar capture‐recapture model, a longer capture history may have helped to better separate these two factors.

Capture probability differed among the five study sites. All three sites in Massachusetts had lower capture probability than those in Maine as well as lower precision in those estimates. Capture probabilities estimated from the Massachusetts sites (0.23–0.33) were similar to those found in other recent studies (0.03–0.35; Villella et al. 2004; Meador et al. 2011; Carey et al. 2019), whereas the estimates from the Maine sites (0.75–0.85) were much higher. The differences among sites may be due to differences in the physical area of the sites. In general, the sites in Massachusetts were larger in area than those in Maine (at least the Pleasant River), and it is possible that the smaller sites received more focused effort. Additionally, the sampling personnel varied by state and observer experience may have been a contributing factor. In Massachusetts, the state natural resource agency (MDFW) collected the data and used seasonal technicians each year to help with sampling. In contrast, the sampling in Maine was contracted to an environmental consulting company (Biodrawversity LLC., Amherst, MA) that specializes in freshwater mussel sampling and whose staff has greater experience on average. Lastly, differences in capture probability may, in part, be related to differences in population density. It is possible that the observation of individuals may result in other nearby individuals being more readily observed, which would theoretically occur more often with increasing density and spatial clustering (Strayer 2008; Haag 2012; Skorupa et al. 2024). While freshwater mussels are frequently observed in patchily distributed populations (Ries et al. 2016), we know of no field study that has formally addressed this issue of spatial clustering with respect to its effect on search rate and individual detection probability.

Brook Floater abundance estimates also differed greatly among sites. Abundance estimates at sites in Massachusetts were significantly lower than those in Maine and were less precise. Given the lack of baseline information, it is possible that those systems differ for a variety of potential reasons, such as poor habitat, the introduction of invasive species (although none of these watersheds contain common invasive bivalves such as *Corbicula fluminea* , *Dreissena bugensis* , or *Dreissena polymorpha* ) or low numbers of host fish in Massachusetts. While surveys of the local fish assemblages were outside the scope of this study, both the Farmington River as well as the Nissitissit River are known to contain diverse fish assemblages including several species known to serve as hosts for Brook Floater. Lastly, the results of this study do not necessarily imply that the Massachusetts sites ever supported as large populations as those in Maine. It is possible that the Massachusetts sites have declined from historical levels; however, it is also possible that they always supported smaller populations due to resource limitations.

While the mean size of individual mussels at most of these sites was similar, this was not the case for the Farmington River, where the mean mussel size was significantly smaller. This result is noteworthy given its contrast to the previous studies in this system. Nedeau and Low (2008) concluded that the Farmington River population was skewed toward larger animals (*n* = 80; mean = 54 mm), and they rarely observed individuals < 40 mm. Our study, however, estimated the mean size to be 44 mm (*n* = 101), and nearly a third of those individuals were < 40 mm, suggesting recent recruitment in that population.

Temporary emigration is one parameter that can be adjusted in the robust‐design model to match the life history characteristics of the study species (Kendall et al. 1997). In our case, we were interested in the possibility of temporary emigration both from horizontal movement upstream/downstream as well as vertical migration (depth in the substrate) related to feeding and reproductive patterns. The “no emigration” scenario was most realistic in the context of horizontal movement due to the sessile nature of mussels and because occasional sampling downstream of some of the study sites did not yield any captures of tagged mussels. The issue of vertical migration was also addressed by including versions of the models that allowed temporary emigration (either random or Markovian). Unlike in some other studies (Meador et al. 2011), the temporary emigration parameter was not useful in our models potentially because of the short timeframe of the sampling seasons, which corresponded to when fertilization is reported to occur in Brook Floater (Nedeau 2008) and all mature individuals are expected to be at the substrate surface (Watters et al. 2001). Temporary (i.e., vertical) migration is likely specific to species and habitats (Watters et al. 2001) and may be affected by seasonal conditions such as droughts which could induce increased burrowing activity (Gough et al. 2012), so these behaviors could be considered for long‐term monitoring studies for other Unionid species.

### 4.2 Implications for Future Sampling Strategies

The differences in demographic parameters observed between sites in Massachusetts and those in Maine suggest that separate strategies may be reasonable for future abundance estimation at these sites. At the Massachusetts sites, more than two secondary periods each year may be necessary to provide reasonable estimates of abundance and allow state managers to detect population declines (or population increases in the event of restoration activity). A minimum of five secondary periods in each of at least three primary periods has been suggested to produce reasonable results (Otis et al. 1978; Pollock 1982); however, that recommendation assumed a capture probability of 0.1, which is considerably lower than the capture probabilities observed in this study (0.23–0.85). In addition, several other factors can affect the appropriate number of secondary periods for a particular study, including the effect size and confidence in the ability to detect an effect, as explained below.

One consideration for sampling frequency is the size of the population trend, whether an increase or decrease in the population, that the resource manager would like to be able to detect. While there is no one‐size‐fits‐all rule for determining a desired level of population change to detect, several factors could be considered depending on the system in question. The manager could consider what actionable steps (e.g., population augmentation/translocation, habitat restoration, or regulation/permitting changes) they would take if a population decline were detected. The manager could also consider the timeframe of those management actions and whether they would be fast enough to restore the population before local extirpation occurred. Here, considerations could be made regarding the minimum viable population (MVP) size. While few examples exist in freshwater mussel literature, some studies have suggested that small populations (~100 individuals) may experience severe genetic bottlenecks (Geist 2010; Geist et al. 2010; Ferreira‐Rodríguez et al. 2019). Related to this, several studies have investigated the Allee effect in populations of freshwater mussels with conflicting results. While some have suggested that low densities of mussels result in significantly reduced reproductive success (Downing et al. 1993), others have found effects of population size on reproductive success only at low current velocities (Terui et al. 2015) or none at all (Mosley et al. 2014).

Sampling frequency is also dependent on the desired level of power to detect an effect. Reviews in past decades found that power analyses were rarely conducted in biological research (Forbes 1990; Rosenfeld and Rockette 1991). More recently, a review found that among 3847 field experiments examining environmental ecosystem stressors, the median power to detect the response magnitude varied from 18% to 38%, suggesting that statistical power remains an underutilized component of research studies (Yang et al. 2022). By tradition, an acceptable level of power given α = 0.05 (Type I error) is 0.8 (1 – β; Cohen 1992; Cohen 1988), although this convention has been disputed more recently. Specifically, Di Stefano (2003) points out that $\frac{\alpha }{\beta }$ = 0.25 implies that a Type I error is four times costlier than a Type II error. In this study, a Type I error would result in managers believing that a population decline has occurred when it has not, while a Type II error would result in managers believing that a population decline has not occurred even though it has. In the instance of a species of conservation concern, this Type II error could be more costly for a manager to commit. Using this logic, a target power of > 0.8 to detect a population decline would likely be preferable. Notably, at small population sizes (50 ≤ *n* ≤ 100) the power to detect even a 50%–60% decline is less than the probability of a Type II error (1—β ≤ 0.5) when only two secondary periods are used. We demonstrate that power could be meaningfully improved by adding one or two additional secondary periods each year (Figure 7). As a result, at small population sizes as many as four or five (or more) secondary periods may be required to detect population losses. To mitigate the potential negative effects of such frequent sampling on the individuals and habitat at the sites (Cope and Waller 1995; Dunn et al. 2000), passive integrated transponder (PIT) tags could be used in place of shellfish tags. These have the benefit of not only reducing handling of individuals but also resulting in higher recapture rates than visual searches alone (Kurth et al. 2007).

In contrast to the scenarios above at sites with low (< 150) population sizes such as our Massachusetts sites, the results seen at the sites in Maine demonstrate that the existing sampling effort by MDIFW is likely sufficient to detect large declines with a high degree of power (> 90%), given the higher abundances and capture probabilities. That said, if abundance is the primary interest, using a simplified estimator such as the Lincoln‐Petersen Index may suffice, although the ability to produce yearly survival estimates would be lost. It may be possible to estimate both parameters (survival and abundance) by alternating periods of multiyear sampling (to estimate survival) with individual years of sampling using closed‐capture models (to estimate abundance). We recognize, though, that multi‐year sampling may not be feasible in many monitoring programs, so considerations can be made on a case‐by‐case basis depending on the specific monitoring objectives and the sampling resources available.

## 5 Conclusions

Estimating demographic parameters is necessary to understand population‐level changes (Klimeš et al. 2022). Estimates of yearly abundance inform natural resource managers of how populations may be responding to recently implemented management actions or environmental stressors (Keever et al. 2025). In the absence of such baseline information, managers may be forced to make less informed decisions, which can result in suboptimal or even ineffective management choices.

While this study was focused on one species of freshwater mussel and only five sites, many of the conclusions drawn could be applied to other species or systems of interest. In particular, the issues of sampling design and appropriate levels of sampling effort are common across ecological studies. Decisions about sampling design and frequency may be difficult to determine initially, especially in a system where the focal species is poorly understood. In these cases, pilot studies (those that may only last a few seasons) can be valuable for understanding where a system may fit along the spectrum of statistical power. Monitoring decisions can be assessed and adjusted as the study progresses informally (Johnson 1999) or formally using an adaptive management framework (Walters and Hilborn 1978; Murray and Marmorek 2003). Although the follow‐up analysis in this study was not the primary objective for the state agencies, we demonstrate the usefulness of considering statistical power to improve sampling designs beyond what was initially considered.

## Author Contributions

**Michael A. Baker:** conceptualization (supporting), data curation (equal), formal analysis (lead), methodology (equal), software (lead), validation (lead), visualization (lead), writing – original draft (lead), writing – review and editing (lead). **Allison H. Roy:** conceptualization (equal), funding acquisition (equal), investigation (supporting), methodology (supporting), project administration (equal), resources (equal), supervision (supporting), writing – review and editing (supporting). **Jason Carmignani:** conceptualization (equal), data curation (equal), investigation (equal), methodology (equal), writing – review and editing (supporting). **Brian J. Irwin:** formal analysis (supporting), methodology (supporting), validation (supporting), visualization (supporting), writing – review and editing (supporting). **Clark Rushing:** formal analysis (supporting), methodology (supporting), validation (supporting), visualization (supporting), writing – review and editing (supporting). **Sean Sterrett:** conceptualization (equal), investigation (equal), methodology (equal), writing – review and editing (supporting). **Beth Swartz:** conceptualization (equal), data curation (equal), funding acquisition (equal), investigation (equal), writing – review and editing (supporting). **Peter D. Hazelton:** conceptualization (equal), formal analysis (supporting), funding acquisition (equal), investigation (equal), methodology (equal), project administration (equal), writing – review and editing (supporting).

## Funding

Funding for this project was provided by the U.S. Fish and Wildlife Service through a multistate Competitive State Wildlife Grant awarded to the Massachusetts Division of Fisheries and Wildlife and the Maine Department of Inland Fisheries and Wildlife. The Georgia Cooperative Fish and Wildlife Research Unit is sponsored jointly by the Georgia Department of Natural Resources, the University of Georgia, the U.S. Fish and Wildlife Service, the U.S. Geological Survey, and the Wildlife Management Institute. The Massachusetts Cooperative Fish and Wildlife Research Unit is sponsored jointly by the Massachusetts Division of Fisheries and Wildlife, the Massachusetts Division of Marine Fisheries, the University of Massachusetts Amherst, the U.S. Fish and Wildlife Service, the U.S. Geological Survey, and the Wildlife Management Institute. Any use of trade, firm, or product names is for descriptive purposes only and does not imply endorsement by the U.S. Government.

## Conflicts of Interest

The authors declare no conflicts of interest.

## Supporting information


**Appendix S1:** Assumptions of capture‐mark‐recapture models using Pollock's robust‐design approach (Pollock 1982) related to sampling of Brook Floater ( *Alasmidonta varicosa* ) at newly established long‐term monitoring sites.

## Data Availability

The authors have made available the following: source code in R statistical language to simulate populations for use in *Objective 2* of the manuscript, the simulated data used in the power analysis described in the manuscript, and the code for conducting population estimates and power analysis. We have also published code used in conducting robust design and Lincoln‐Petersen estimation techniques on populations in Maine and Massachusetts (*Objective 1*). These data and associated code are currently located on a public Zenodo repository operated by the corresponding author (https://doi.org/10.5281/zenodo.19074904). We will not be publishing raw data collected from mussel capture‐mark‐recapture surveys as these data belong to the Maine Department of Inland Fisheries and Wildlife, and the Massachusetts Division of Fisheries and Wildlife. Interested parties may contact these agencies directly for access to those data.
