# Supplementary material for: Using Demographic Estimation and Power Analysis to Inform Monitoring Efforts and Detect Declines in Freshwater Mussels
Source: Ecol Evol. 2026 May 14;16(5):e73532. doi: 10.1002/ece3.73532 (PMC13175712; doi:10.1002/ece3.73532)
Supplement: Supplementary file 1 — Appendix S1: Assumptions of capture‐mark‐recapture models using Pollock's robust‐design approach (Pollock 1982) related to sampling of Brook Floater ( Alasmidonta varicosa ) at newly established long‐term monitoring sites. [file ECE3-16-e73532-s001.docx]

Appendix S1. Assumptions of capture-mark-recapture models using Pollock’s robust-design approach (Pollock 1982) related to sampling of Brook Floater (*Alasmidonta varicosa*) at newly established long-term monitoring sites.

1) The population is demographically closed among secondary periods (individual sampling occasions within a season). Because sampling took place during a short period within the summer months, we assumed that any births/deaths or movement into/out of the populations was negligible.

2) The entire population of interest is subject to capture. This can be split into two parts, both of which deal with the issue of detectability. First, we recognize that searching for mussels in the substrate using snorkel methods is biased toward capturing larger individuals because of the impracticality of locating smaller mussels. As a result, all abundance estimates derived from the models below represent the sampleable portion of the population. While no single cut-off has been proposed related to freshwater mussel sampling, the results of several studies suggest that individuals <20 mm likely will not be detected using snorkel surveys alone without constructing quadrats and sifting sediment (Obermeyer 1998; Smith et al. 2001; Villella et al. 2004; Inoue et al. 2014). Secondly, we recognize that a portion of the population may at any given time not be susceptible to detection either because of horizontal temporary emigration or because they are completely buried in the sediment (vertical migration). To test this assumption, versions of the models included each type of temporary emigration (no emigration, random emigration, or Markovian emigration; Meador et al. 2011). Additionally, all sampling took place during short periods in the summer months when all individuals were expected to be at the surface of the substrate (Watters et al. 2001; Nedeau 2008).

3) No tag loss or misidentification of individuals occurs. To mitigate the effects of tag loss, all individuals were double tagged, one on each valve. Previous studies have reported low tag loss (1–5% over 3–6 years; Lemarié et al. 2000; Wisniewski et al. 2013) in freshwater mussels leading us to assume that the probability of double tag loss on a given individual is negligible for our study.

4) Fates of individuals with respect to survival and capture probability are independent of one another. While studies have demonstrated variable survival across habitat types within a particular reach and across changing habitats over time (Hamilton et al. 1997; Inoue et al. 2014), no research has investigated whether yearly survival of an individual mussel is directly linked to that of another individual. With regard to capture probability, we recognize the potential for the detection of an individual to affect the search efficiency and search rate of the observer in such a way that other adjacent individuals may be more easily detected. This could occur either due to the spatial clustering of individuals or the overall number of individuals at a site. Green and Young (1993), however, found that at low densities (< 0.1/m2) individual mussels were statistically randomly distributed following a Poisson distribution (Smith et al. 2003; Smith 2006). We are aware of no other work that has attempted to address the effects of density or spatial clustering on detection in these systems. As a result, we assumed that individual detections were independent.

5) Tagging an individual does not affect its survival. No studies have demonstrated reduced survival for tagged freshwater mussels; therefore, we assumed that tagging was not a source of mortality.

6) Capture probability among secondary periods is constant unless specified in the model. Models were included that allow capture probability to vary across secondary periods due to factors such as occasion specific habitat characteristics and environmental conditions at the time of sampling.

7) Survival probability among primary periods is constant unless specified in the model. Models were included that allow survival to vary across primary periods due to factors such as year specific population age structure or acute mortality events.

8) Capture probability and recapture probability are shared unless specified in the model. Because we were not using passive integrated transponder tags to mark the mussels, there is no reason to believe that the presence of a tag would improve the recapture probability. Additionally, due to the sessile nature of mussels, it would not be possible for them to exhibit a behavioral response to capture.

REFERENCES

Green, R.H., and Young, R.C. 1993. “Sampling to detect rare species.” Ecological Applications 3:351–356. https://doi.org/10.2307/1941837

Hamilton, H., Box, J.B., and Dorazio, R.M. 1997. “Effects of habitat suitability on the survival of relocated freshwater mussels.” Regulated Rivers: Research and Management 13:537–541. https://doi.org/10.1002/(SICI)1099-1646(199711/12)13:6<537::AID-RRR483>3.0.CO;2-Y

Inoue, K., Levine, T.D., Lang, B.K., and Berg, D.J. 2014. “Long-term mark-and-recapture study of a freshwater mussel reveals patterns of habitat use and an association between survival and river discharge.” Freshwater Biology 59: 1872–1883. https://doi.org/10.1111/fwb.12389

Lemarié, D.P., Smith, D.R., Villella, R.F., and Weller, D.A. 2000. “Evaluation of tag types and adhesives for marking freshwater mussels (Mollusca: Unionidae).” Journal of Shellfish Research 19(1): 247–250.

Meador, J.R., Peterson, J.T., and Wisniewski, J.M. 2011. “An evaluation of the factors influencing freshwater mussel capture probability, survival, and temporary emigration in a large lowland river.” Journal of the North American Benthological Society 30(2): 507–521. https://doi.org/10.1899/10-105.1

Obermeyer, B.K. 1998. “A comparison of quadrats versus timed snorkel surveys for assessing freshwater mussels.” The American Midland Naturalist 139(2): 331–339. https://doi.org/10.1674/0003-0031(1998)139[0331:ACOQVT]2.0.CO;2

Pollock, K.H. 1982. “A capture-recapture design robust to unequal probability of capture.” Journal of Wildlife Management 46(3): 752–757. https://doi.org/10.2307/3808568

Smith, D.R. 2006. “Survey design for detecting rare freshwater mussels.” Journal of the North American Benthological Society 25(3):701–711. https://doi.org/10.1899/0887-3593(2006)25[701:SDFDRF]2.0.CO;2

Smith, D.R., Villella, R.F., and Lemarié, D.P. 2001. “Survey protocol for assessment of endangered freshwater mussels in the Allegheny River, Pennsylvania.” Journal of the North American Benthological Society 20(1): 118–132. https://doi.org/10.2307/1468193

Smith, D.R., Villella, R.F., and Lemarié, D.P. 2003. “Application of adaptive cluster sampling to low-density populations of freshwater mussels.” Environmental and Ecological Statistics 10:7–15.

Villella, R.F., Smith, D.R., and Lemarié, D.P. 2004. “Estimating survival and recruitment in a freshwater mussel population using mark-recapture techniques.” American Midland Naturalist 151: 114–133. https://doi.org/10.1674/0003-0031(2004)151[0114:ESARIA]2.0.CO;2

Wisniewski, J.M., Shea, C.P., Abbott, S., and Stringfellow, R.C. 2013. “Imperfect recapture: a potential source of bias in freshwater mussel studies.” American Midland Naturalist 170: 229–247. <https://doi.org/10.1674/0003-0031-170.2.229>
